# Telehealth for the Provision of Occupational Therapy: Reflections on Experiences During the COVID-19 Pandemic

**DOI:** 10.5195/ijt.2020.6328

**Published:** 2020-12-08

**Authors:** Sue Dahl-popolizio, Heidi Carpenter, Melissa Coronado, Nicholas J. Popolizio, Connor Swanson

**Affiliations:** 1 Arizona State University, Phoenix, Arizona, USA; 2 Banner Health, Tucson, Arizona, USA; 3 Northern Arizona University, Phoenix, Arizona, USA; 4 Boston College, Boston, Massachusetts, USA

**Keywords:** COVID, Occupational therapist, Occupational therapy, Telehealth, Telerehabilitation

## Abstract

During the COVID-19 pandemic of 2020, healthcare professionals worldwide abruptly shifted from an in-person to a telehealth service delivery model. Many did so without advanced training or preparation. This cross-sectional study explored how occupational therapy practitioners (OTPs) used telehealth during the COVID-19 pandemic, and whether they found it to be an effective service delivery model that should be a permanent option for providing occupational therapy services. An online survey was disseminated; it included Likert scale questions, multiple option questions, and open-ended questions regarding telehealth use during the COVID-19 pandemic. Of the 230 respondents, 176 (77%) support telehealth as a substitute for in-person services; 179 (78%) support telehealth as a permanent option for occupational therapy service delivery. This information lends support to the uninterrupted use of telehealth by OTPs when government emergency orders in response to COVID-19 expire.

As the 2020 COVID-19 pandemic intensified, many healthcare professionals were required to abruptly transition their customary in-person treatment to telehealth, often without advance preparation or training. These practitioners are now uniquely poised to provide insights regarding the benefits and limitations of rapidly deployed telehealth. The purpose of this cross-sectional study was to explore how such occupational therapy practitioners (OTPs) are using telehealth, and whether they find it an effective service delivery model.

The American Occupational Therapy Association's (AOTA, 2018) use of the term ‘telehealth' is inclusive of evaluation, intervention, consultation, supervision, and remote monitoring provided by OTPs across practice settings.

## IMPACT OF TELEHEALTH ACROSS HEALTHCARE

Telehealth has been slowly gaining traction as a service delivery model across healthcare professions worldwide (Smith et al., 2020). Telehealth has been shown to be effective in improving outcomes in both physical and behavioral health conditions (Kruse et al., 2017). While published reports have supported the use of telehealth, many have expressed the need for further evaluation to identify “best practices” (Smith et al., 2020; Wosik et al., 2020).

Cost, distance, and time are known barriers to healthcare access for patients, especially after an initial healthcare office visit or treatment (Dirnberger & Waisbren, 2020; Powell et al., 2017). Telehealth can save substantial time and money, provides more convenient access to care, and has been met with overwhelmingly positive feedback from patients (Dirnberger & Waisbren, 2020; Morony et al., 2017; Powell et al., 2017). Patients who received care via telehealth reported high rates of satisfaction with telehealth and expressed interest in attending future visits remotely (Cason, 2014; Dirnberger & Waisbren, 2020; Kruse et al., 2017; Powell et al., 2017). Telehealth also adds a convenience and comfort factor for patients, as they can participate in OT sessions from their home, on their own device, and attend the visit with whomever they choose to be present with them (Dirnberger & Waisbren, 2020; Morony et al., 2017; Powell et al., 2017). Telehealth increases access to care for those who would otherwise have limited or no access due to travel, inclement weather, limited transportation, and other access barriers (AOTA, 2018; Cason, 2014; Dirnberger & Waisbren, 2020; Kruse et al., 2017; Powell et al., 2017; Wallisch et al., 2019).

Many healthcare providers have successfully used telehealth to deliver services including physicians across specialties, psychologists, nurses, OTPs, physical therapy practitioners, and speech language pathologists (Cason, 2014; Dirnberger & Waisbren, 2020; Kruse et al., 2017; Morony et al., 2017; Powell et al., 2017; Wallisch et al., 2019). Surgeons have used telehealth as a substitute for in-person follow-up appointments, especially when time and distance were limiting factors for their patients (Dirnberger & Waisbren, 2020). Nurses have used telehealth to answer questions and ensure the understanding of the patient via “teach back,” meaning the patient either demonstrates or explains the instructions they received. This method has been found to be especially helpful for patients with low health literacy and/or socio-economic status (Morony et al., 2017). Telehealth has also been used to provide remote urgent and non-urgent nursing services and to provide instructions and advice in a helpline format (Fathi et al., 2017; Morony et al., 2017; Powell et al., 2017). Primary care physicians have used telehealth for routine visits with established patients where time, money, work absenteeism, and mobility were obstacles (Powell et al., 2017). OTPs use synchronous and asynchronous methods of telehealth to help patients modify their environments, routines, and habits, as well as to develop skills and strategies to participate in meaningful activities (AOTA, 2018).

With effective preparation and communication between the patient and the healthcare practitioner, many interventions can be provided using telehealth. Patient helplines, education and teach-back, office visits, post-op follow up care, remote management of communicable diseases, and synchronous and asynchronous monitoring of conditions are evidence-based applications of telehealth (Dirnberger & Waisbren, 2020; Kruse et al., 2017; Morony et al., 2017; Smith, 2020; Wosik, 2020). OTPs have used telehealth to help their patients develop skills, habits, and routines, improve their patients' health status, modify their environments, and teach techniques and strategies to maximize self-management and patients' independence (AOTA, 2018).

Information and communication technologies used in telehealth include telephone, video (with audio), electronic gaming systems, sensor technologies, digital cameras, email, and more. These technologies have been used to provide OT services synchronously and asynchronously through telehealth (AOTA, 2018; Dahl-Popolizio et al., 2014; Dirnberger & Waisbren, 2020; Morony et al., 2017; Powel et al., 2017; Wosik et al., 2020). The diversity of available technologies that can support the delivery of services through telehealth increases access to care.

The audio-only telephone (versus the “smart” mobile cell phone with video capability), has historically been devalued due to the lack of visual feedback. However, as it is low tech and ubiquitous, the audio-only telephone can increase access to care and is the most accessible technology for telehealth across the socioeconomic continuum (Wosik et al., 2020). The audio-only telephone, in addition to other telehealth technologies, has been an effective means of providing education, health-related advice, and urgent and non-urgent care.

## THE USE OF TELEHEALTH TO DELIVER OCCUPATIONAL THERAPY SERVICES

Telehealth can be used by OTPs for evaluation, intervention, education, and to prevent injury or exacerbation of conditions (AOTA, 2018; Cason, 2015). Telehealth facilitates collaboration and consultation with other professionals, which facilitates coordination of care (AOTA, 2018; Cason, 2014; Cason & Jacobs, 2014). OTPs are implementing telehealth across many practice settings including in early intervention, schools, pediatric private practice, hospitals, burn units, productive aging, workplace ergonomics, mental health, and inpatient and outpatient settings (Cason & Jacobs, 2014).

In the school setting, telehealth has been shown to increase timely access to care, and provide care to students who could not attend in-person therapy sessions (AOTA, 2018; Cason, 2014; Rortvert & Jacobs, 2019). Benefits of telehealth in the school setting include cost-savings, flexible scheduling, and the ability to provide services to homebound students (Rortvert & Jacobs, 2019). School-based OTPs are effectively using telehealth for caregiver coaching, to enhance children's ability to follow directions and improve social skills, and to address children's complex medical needs including motor control issues, feeding disorders, and issues related to autism spectrum disorder (AOTA, 2018; Langbecker, 2019). Parents, caregivers and patients are often more engaged as a team with the OTP when receiving services via telehealth than when receiving occupational therapy services in-person (Wallisch et al., 2019). Additionally, telehealth promotes increased access to care by enabling therapists to use time that would normally be spent commuting to see more patients, and for patients in rural areas to have increased access to therapy services (Langbecker, 2019; Rortvert & Jacobs, 2019).

OTPs currently use telehealth for wheelchair assessments, home visits, orthopedic consultations, activities of daily living (ADL) assessments, hand function assessments, mobility and adaptive equipment assessments and training, and more (Cason, 2015). OTPs contribute directly to population health as they are trained to address patient factors, performance skills and patterns, contexts and environments, and the activity demands that affect health and engagement in occupations, including behavioral health and behaviors that affect health (AOTA, 2014; Cason, 2015; Dahl-Popolizio et al., 2017). OTPs can contribute to population health via telehealth by providing interventions facilitating self-management of chronic conditions, behavioral health issues, and working toward implementation of behavioral health screenings, coordination of care, and health and wellness (Cason, 2015).

The purpose of this study was to determine whether OTPs who have used telehealth assess this service delivery model as being effective and sustainable. The researchers sought to answer the following questions: How are OTPs using telehealth? Do OTPs find telehealth to be a satisfactory and effective service delivery model? Study results can inform advocacy efforts at the national and state levels regarding continued use of telehealth and reimbursement after COVID-related emergency orders expire. In addition, the results can be used to guide telehealth best practices and identify areas that require further research.

The authors anticipated that OTPs using telehealth would identify specific diagnoses that could be treated and interventions that could be provided via telehealth. They also projected that OTPs would report on whether telehealth was an effective service delivery model for occupational therapy services, their satisfaction with telehealth, if treatment goals could be met via telehealth, if attendance was improved, and if the OTPs obtained reimbursement for OT services provided through telehealth.

## METHODS

### DESIGN

This exploratory study used a cross-sectional design with a web-based survey to gather data. Researchers chose this format to maximize the number of respondents by allowing the researchers to access practitioners across the United States. Google Forms® was the platform used for this study. The Arizona State University Institutional Review Board determined this study exempt after review.

### PROCEDURES

Prospective participants received an introductory email with a link to the Google Forms® survey. The respondents' submission of the survey was acknowledgement of their consent to participate in the study. Respondents were recruited via professional association listservs and social media. They were asked to answer a survey comprising five demographic and background questions, and 15 questions regarding their experience with, and perceptions of providing OT services through telehealth. The 15 non-background questions were a combination of Likert scale and short answer questions. The president of the Arizona Occupational Therapy Association (ArizOTA) emailed the survey link to the other 49 state occupational therapy association presidents and asked each to post it on their member listserv. A reminder was sent to this same group one week later. The researchers also posted the survey link to the survey page on the American Occupational Therapy Association's (AOTA) CommunOT group forum, and to several relevant Facebook® groups. There was no follow up with these social media groups. The survey was open for three weeks, from May 26 to June 14, 2020. Due to the use of social media, it is impossible to determine an accurate response rate. Responses from respondents who completed all the required questions were included in the survey data collected, and in the analysis. Only the last open-ended question was optional. This question asked for any additional information the respondent would like to add; not all respondents answered that question.

### PARTICIPANTS

**Figure 1:**
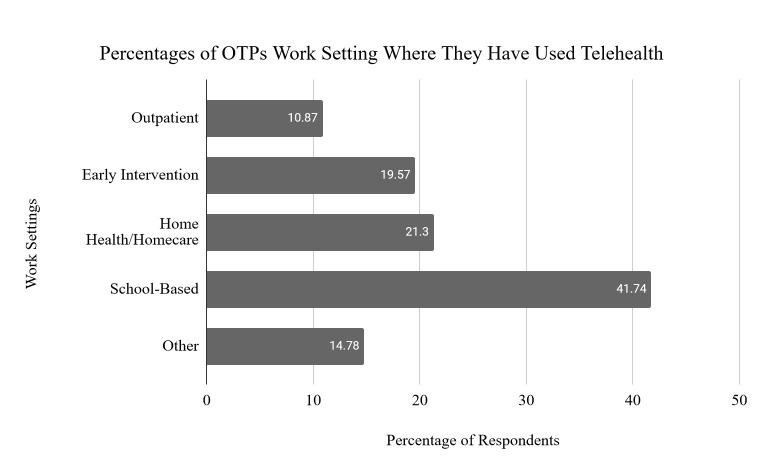
Percentages of OTPs Work Setting Where They Have Used Telehealth

*Note.* Other settings included acute care, inpatient, pediatric, skilled nursing facility, and research.

Occupational therapists and occupational therapy assistants practicing across the United States who were currently using, or who had used, telehealth were the researchers' target population. These professional designations are reflected collectively in the term occupational therapy practitioners or OTPs throughout this article. The demographic questions determined that of the 230 total respondents, 191 (83%) were occupational therapists, and 39 (17%) were occupational therapy assistants working as OTPs across 32 of the 50 states. The primary work settings of these OTPs are reflected in Figure 1.

### SURVEY INSTRUMENT

Prior to administering the survey, the research team held a focus group of OTPs currently using telehealth. This focus group was comprised of nine OTPs and one OTA. All the OTPs had experience using telehealth, either long term or as a result of the COVID-19 pandemic and related government orders that mandated OTPs to provide services via telehealth. The researchers asked the participants about their experiences and what information would be helpful to collect in order to continue using telehealth in their practices. As there were no existing validated surveys to gather the information that the researchers sought, the researchers developed survey questions informed by the participants' responses. Demographic information included: license type (OTR or OTA), state and setting of primary practice, populations or conditions treated via telehealth, and telehealth technologies/platforms used. The rest of the survey included forced-choice Likert scale questions, multiple option answers (with instructions to check all that apply), and open-ended short answer questions to allow the respondents to provide detailed information that was specific to their experiences. The Likert scale answer choices to the statements regarding telehealth were: *strongly agree, agree, neutral, disagree, and strongly disagree* (see Appendix for the survey questions)*.* The short answer questions encouraged respondents to list diagnoses of patients that they had treated, and the interventions they provided via telehealth, as well as any other information regarding their experiences that they felt would be relevant in determining if telehealth was an effective, sustainable service delivery model for OTPs. The instructions preceding the questions were brief and instructed the OTPs to assume that telehealth was appropriate for the patient's therapeutic needs and that the patient had the necessary technology to receive treatment via telehealth.

### DATA ANALYSIS

Following the close of the survey, data collected through Google Forms was downloaded by the researchers and analyzed by the Arizona State University Biostatistics Core Team. The team eliminated duplicate answers. Descriptive statistics, including counts and percentages, were calculated for all the questions. Two members of the research team thematically categorized the open-ended responses, with a third member acting as arbitrator where there was disagreement. Using IBM SPSS 24, the team analyzed the Likert scale questions and the thematically grouped open-ended responses with counts and percentages. The final open-ended question was analyzed using grounded theory by constant comparative analysis to identify themes that emerged from the respondents' reported lived experiences and perspectives of using telehealth for occupational therapy service delivery. Two researchers independently examined the responses, identified and defined the themes, and used a consensus coding approach to determine which themes reflected the response *(0=theme was not present; 1=theme was present)*.

## RESULTS

To address the goal of determining how OTPs are using telehealth and whether they find it to be a satisfactory and effective service delivery model, the researchers used the counts and percentages of the responses to the forced choice Likert scale questions, the multiple option questions, and the open-ended questions. The researchers first analyzed the 230 forced responses to the Likert scale questions, multiple option, and open-ended questions. When asked if telehealth should be a service delivery model that is offered permanently, 176 (77%) of respondents supported telehealth as a substitute for in-person clinical visits, and 179 (78%) supported telehealth as a permanent option to be used in addition to in-person visits. The large number of responses supporting both options suggests that many therapists feel that telehealth should be a service delivery option for occupational therapy services. See Table 1 for the specific questions asked in the Likert scale format and responses to each question.

**Table 1:** Specific Questions Asked in the Likert Scale Format and Responses

Question	Strongly Disagree		Disagree		Neutral		Agree		Strongly Agree	
	Count	%	Count	%	Count	%	Count	%	Count	%
In my professional opinion, telehealth is an effective platform for the delivery of OT services	12	5	21	9	40	17	104	45	53	23
Telehealth should be a treatment platform option for the delivery of OT services permanently for those conditions that can be treated successfully via telehealth	8	3	23	10	21	9	71	31	107	47
I had fewer no-shows for telehealth visits than I usually have for in-person visits	37	16	48	21	47	20	45	20	53	23
I was able to achieve established patient goals via telehealth	12	5	27	12	43	19	96	42	52	23
I achieved similar health outcomes using telehealth as I would have expected in person	25	11	61	27	41	18	65	28	38	17
I was able to be sufficiently productive using telehealth	12	5	33	14	37	16	92	40	56	24
I was satisfied with telehealth as a delivery platform	21	9	33	14	35	15	84	37	57	25
I would recommend telehealth as a service delivery platform to my friends and family members	16	7	35	15	44	19	67	29	68	30
Patients were satisfied with telehealth as a delivery platform	8	4	29	13	55	24	81	36	53	23
Caregivers were satisfied with telehealth as a delivery platform	12	5	28	13	41	18	96	43	47	21

*Note.* OT = occupational therapy.

**Figure 2:**
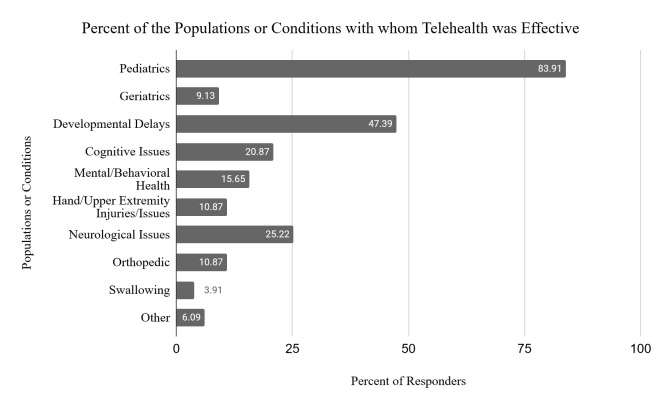
Percent of the Populations or Conditions with whom Telehealth was Effective

**Figure 3:**
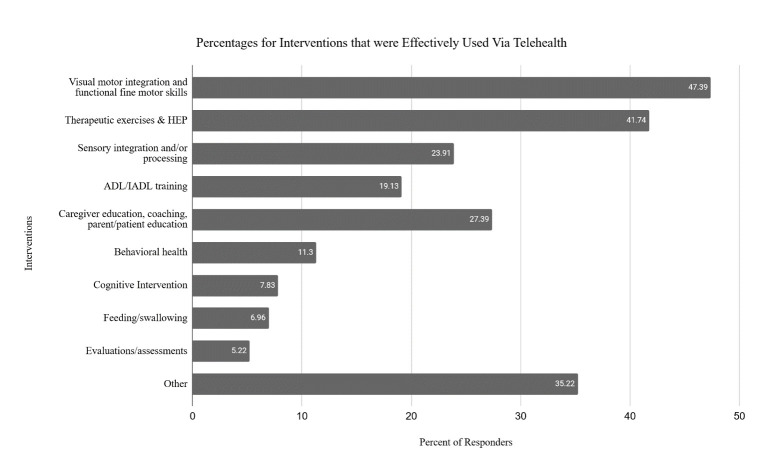
Percentages for Interventions that were Effectively Used via Telehealth

*Note.* Other interventions included: play/leisure, visual perceptual, Ziteboard (whiteboard) activities targeting a variety of skills, and safety training.

Participants provided up to three responses indicating what populations or conditions they were effectively able to treat, and what interventions they could effectively provide via telehealth. See Figure 2 for the most common populations and conditions and Figure 3 for the most common interventions provided through telehealth.

**Figure 4:**
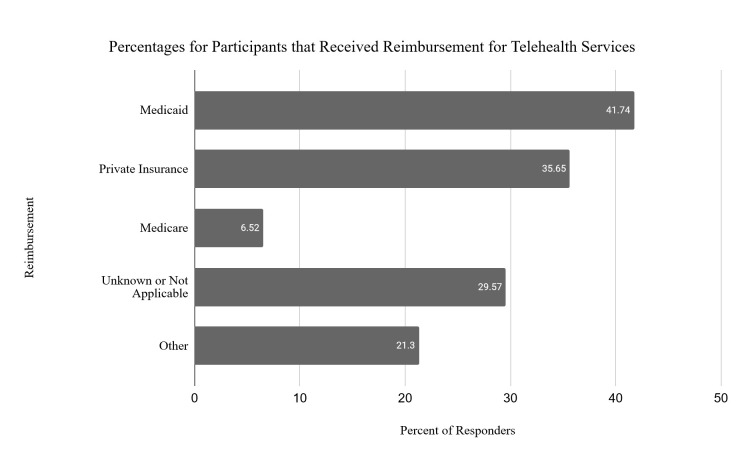
Percentages for Participants that Received Reimbursement for Telehealth Services

*Note.* Other reimbursement included: school districts, worker's compensation, state-funded early intervention programs, regional centers, private pay, pediatric programs, grant reimbursement, and various governmental agency funds.

As reimbursement concerns have been identified in the literature as a barrier to adopting telehealth, one of the survey questions asked the respondents if they received reimbursement for the services they provided via telehealth. See Figure 4 for the breakdown of third-party payors. Approximately one-third of respondents did not know their source of reimbursement or stated that this question was not applicable. These therapists likely did not bill directly for their services.

**Figure 5:**
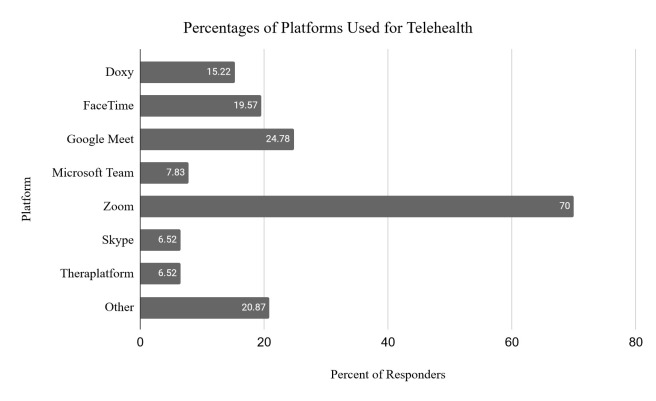
Percentages of Platforms Used for Telehealth

*Note.* Other platforms included Curatess, Vsee, Adobe Connect, Google Duo, phone, InTouch, HealthCare Anywhere, MiCHART, Blackboard, Whatsapp, WebEx, and Facebook Messenger.

Respondents were asked which telehealth platforms they had used to deliver services. Most OTPs reported that they used a synchronous video platform (e.g., Zoom, Doxy, Google Meet, Skype, Microsoft Team). Other interactive platforms, such as FaceTime were used as well as smart phones for audio, text, and instant messaging. For some of these functions, a smart phone is required, though the type of phone was not identified. Some respondents reported using an interactive tool or program embedded in electronic health record systems. See Figure 5 for specific telehealth platforms used by OTPs.

The final open-ended question asked the respondents for any additional information they would like to share regarding their experience with, and perceptions of, telehealth. Using an inductive approach, the researchers ultimately categorized the 171 responses to this question into seven themes. To provide context for this frequency analysis, exemplar responses have been provided for each theme with the response count and percentage breakdown:

*Technical issues* (n=18; 7.83%). This theme addresses the multiple issues that can arise with technology and telecommunication.

“Often technical problems would occur such as audio/visual not working, internet lagging or dropping, and computers requiring updates at time of session.”

While this theme comprised fewer responses, it was mentioned often enough to include technical issues as a significant theme. It suggests confronting technical obstacles is necessary to provide effective occupational therapy services.

*Lack of personal contact* (n=29; 12.61%). Some practitioners identified a lack of personal contact as a significant barrier when providing occupational therapy services via telehealth. At the same time, others suggested there was a positive aspect as it increased access to care in general, and in situations where the patient could not attend therapy services in-person.

“I was greatly missing my clinical hands to facilitate ‘wanted' body movement and or posture, bimanual approaches and [it was] difficult to explain how to prepare an activity to maximize effectiveness.”

“It is not a full substitute for in-person interventions, however I am able to see a lot more clients in a working day.”

“It is just better to be there in person. However, I hope we can still use it as an option during bad weather, a family member is sick and can't get into a clinic, etc.”

*Not effective with all populations* (n=62; 26.96%). This theme references the ability to adequately provide services with specific patient populations. When using telehealth, some practitioners expressed that working with certain populations was difficult. However, shifting from a direct intervention to a caregiver coaching model may address these concerns.

“I work with students with significant cognitive impairments and autism. It was challenging to be effective with this clientele.”

“Difficult for wound management, difficult for neurological patients, difficult for cognitive retraining.”

“Very difficult with early intervention and preschool age.”

Environment and engagement also impacted the effectiveness and further demonstrated the barriers that existed with some populations.

“Kids won't stay on camera;”

“Lacked quiet space or a place to work.”

*Parent/caregiver involvement improves effectiveness* (n=61; 26.52%). The transition to telehealth caused a shift in the role that many parents/caregivers played in the therapy process. Prior to telehealth, many parents/caregivers did not play an active role in the therapy session, as the practitioner led the session with the patient either in the clinic or in the home. With telehealth, parents/caregivers were required to engage in the session to ensure client participation.

“As parents are often needed to assist their child during teletherapy, they are learning strategies for supporting their children as well.”

Some practitioners found that telehealth facilitated parent/caregiver engagement.

“Parents cannot ‘hide' as easily when it comes to their follow through.”

To further support this theme, another practitioner expressed:

“Families felt more empowered because they were more involved during sessions.”

*Effective for occupational therapy delivery* (n=96; 41.74%). This theme addressed how services were delivered effectively using a telehealth service delivery model. It was the most prevalent theme throughout the open-ended responses.

“Telehealth provides an increased level of generalization of skills for the client and more skill acquisition for the caregivers to carry over effectively outside of treatment.”

Others identified that “Coaching was more effective virtually;” and “I felt telehealth was a very effective form of treatment for most of the kiddos on my caseload.”

Phrases such as, “very helpful in assessing home environment,” and “telehealth saves time and money” reflect some OTPs' views about additional ways that telehealth is effective for delivering occupational therapy services.

*Increases access to care* (n=29; 12.61%). This theme identifies how using telehealth can increase access to care.

As one OTP stated, “It [telehealth] fills the gap of finding rural therapists, decreases cost.”

Another respondent said, “Telehealth allows for children in underserved areas to receive private therapy just as their peers in more resource abundant areas can.” Another OTP echoed the perspective that telehealth enables rural students to receive occupational therapy services.

“We have a high percentage of online charter schools as well as rural areas in which teletherapy is the only way to deliver OT services. It truly is an essential service!”

*Telehealth should be a permanent option for patients/caregivers* (n=37; 16.09%). This theme addresses one of the primary aims of the study, to assess support for the continued use of telehealth after emergency orders have expired. Practitioners expressed support for the continued and permanent use of telehealth due to the effectiveness of the service delivery model and the progress their patients achieved:

“Telehealth has been an incredible tool in the online charter school environment. Families, students, and educators have very positive feedback for us and survey research we completed indicated that the preference was for telehealth services over in-person.”

“One parent told me that her son made more progress during the 6 weeks of telehealth than he had during the entire school year.”

“Teletherapy is extremely effective and should remain an option permanently.”

## DISCUSSION

These results answer the research questions and suggest that OTPs using telehealth find it to be a satisfactory and effective service delivery model.

The occupational therapy practitioners surveyed had a vast range of experiences using telehealth, with positive responses outweighing negative. For most questions, OTPs' responses varied greatly, even within the same home-based setting and population. For example, some pediatric therapists had more “no shows” while some had fewer. Some therapists felt early intervention services could not be provided effectively with telehealth. Other practitioners felt their outcomes were better as a result of using telehealth for early intervention services. The caregiver coaching model is considered best practice whether services are provided in-person or through telehealth, and the use of telehealth facilitated the use of a caregiver coaching model (Wallisch et al., 2019).

Perhaps these differences can be attributed to parental responses to challenging circumstances in their home environments. Many parents had other children at home, work commitments, or activities interfering with their participation in the OT session; in these situations, parent engagement may have been limited. Other OTPs experienced better outcomes when parents facilitated their children's participation in the OT session. This variation in home settings could explain why, when asked if the OTPs achieved similar outcomes with telehealth compared to in-person visits, the number of positive versus negative responses was similar. Though there were more positive responses with 103 (45%) either agreeing or strongly agreeing with the statement that telehealth was an effective service delivery model, the number of negative responses was similar with 86 (37%) disagreeing or strongly disagreeing; 41(18%) were neutral.

The Likert scale questions were used to determine OTPs opinions on the use and sustainability of telehealth. The questions were written in a manner to determine a positive or negative perception of telehealth. *Agree* and *strongly agree* responses reflected a positive attitude to, or experience with, the use of telehealth; *disagree* and *strongly disagree* indicated negative perceptions of telehealth. Responses of *neutral* were tracked as well and interpreted as neither positive nor negative.

Over 50% of the participants agreed or strongly agreed to all but two of the questions indicating that OTPs were receptive to the use of telehealth as a service delivery model for OT services. Regarding whether telehealth was an effective service delivery model, 153 (68 %) strongly agreed or agreed, 33 (14%) strongly disagreed or disagreed, and 40 (17%) were neutral. Whether telehealth should be a permanent option for delivery of occupational therapy services, 178 (77%) strongly agreed or agreed, 31(13%) strongly disagreed or disagreed, and 21 (9%) were neutral. When asked if OTPs were able to effectively achieve patient goals via telehealth, responses indicated 148 (64%) strongly agreed or agreed, 39 (17%) strongly disagreed or disagreed, and 43 (19%) were neutral. When asked if OTPs were sufficiently productive using telehealth, 148 (64%) strongly agreed or agreed, 45 (20%) strongly disagreed or disagreed, and 37 (16%) were neutral. Most respondents indicated that they were satisfied with telehealth as a service delivery model, with 141 (61%) strongly agreeing or agreeing, 54 (23%) strongly disagreeing or disagreeing, and 35 (15%) neutral. Of the total respondents, 135 (59%) strongly agreed or agreed, 51 (22%) strongly disagreed or disagreed, and 44 (19%) were neutral to the question of whether they would recommend telehealth as a service delivery model to a friend or family member. In response to the question of whether patients were satisfied with OT services delivered through telehealth, 134 (58%) strongly agreed or agreed, 37 (16%) strongly disagreed or disagreed, and 55 (24%) were neutral. Regarding whether caregivers were satisfied with telehealth as a service delivery model, 143 (62%) strongly agreed or agreed, 40 (17%) strongly disagreed or disagreed, and 41 (18%) were neutral. Except for the questions regarding whether no-shows, and whether outcomes obtained via telehealth were similar to in-person outcomes, there was consensus towards agreement (with agree and strongly agree responses greater than 50%). When asked if no-show rates decreased with telehealth, 98 (43%) agreed, 85 (37%) disagreed, and 47 (20%) were neutral.

The overall responses to both the Likert scale questions and the open-ended questions indicate that most OTPs are satisfied with telehealth and perceive telehealth as an effective service delivery model for OT services. With nearly 80% of respondents answering positively to the questions regarding whether telehealth can be used as a substitute for in-person visits, or as an option to use in addition to in-person visits, OTPs appear to be receptive to telehealth as a permanent treatment option.

The themes that emerged provide a real-world perspective of OTPs delivering services via telehealth. Of the seven overarching themes, three may be perceived with a more negative slant (i.e., technical issues, lack of personal contact, not effective with all populations) while the remaining four appear to reflect a more positive view (i.e., parent/caregiver involvement improves effectiveness, effective for occupational therapy delivery, increases access to care, telehealth should be a permanent option for patients/caregivers). Generally, these themes present topics that should be considered when using telehealth and may provide insight for developing future training programs for OTPs to maximize the effectiveness of this service delivery model and improve the experience for patients and caregivers served through telehealth technologies.

Some limitations to this study include the small sample size and the uneven distribution of responses across states. There were 230 respondents in relation to the approximately 137,000 OTPs in the United States (AOTA, 2010). There were variations in responses across states with 164 of the 230 responses represented by four states. Arizona (86), North Carolina (37), Texas (26), and New York (15) had the highest response rates. Other states had five or fewer respondents, with only 32 of the 50 states represented in the responses. Because the laws governing the use of telehealth currently vary across states; information across all US states is required to generalize the study results.

Another limitation was the lack of differentiation between OTPs who had experience and had developed skills to use telehealth effectively and those who had no experience and were not familiar, or comfortable with the telehealth prior to the COVID-19 pandemic. Although the researchers asked for additional information that the OTPs thought would be helpful, there was no guidance regarding what information the OTPs should include. As a result, the responses may have been biased against or in favor of telehealth. For example, participants may have been more likely to respond if they were frustrated with the service delivery model, or were invested in ensuring telehealth remains a permanent option for OT service delivery. Additionally, this study did not explore the OTPs' perceptions of telehealth based on their comfort with using technology, which may also impact OTPs' experiences and responses. These limitations should be addressed in future studies.

The outcomes of this study support the continued use of telehealth by OTPs as an effective service delivery model. These findings are consistent with ongoing US and state level efforts to continue OT telehealth-based practice after emergency orders related to the COVID-19 crisis are lifted.

## APPENDIX

**Demographic Questions:**

Which state do you primarily practice in (drop down menu - check one)?

Please select your credential:

- Occupational Therapist
- Occupational Therapy Assistant

Select your work setting where you have used telehealth (check all that apply)

- School-Based
- Acute Care
- Inpatient Rehabilitation
- Outpatient
- Homecare
- Home Health
- Skilled Nursing Facility
- Early Intervention
- Other:

Select the populations and/or conditions with whom you have used telehealth (check all that apply)

- Pediatrics
- Geriatrics
- Hand/Upper Extremity Injuries/Issues
- Mental/Behavioral Health
- Neurological Issues
- Developmental Delays
- Orthopedic
- Cognitive
- Swallowing
- Other:

What platform(s) have you used for telehealth? (check all that apply)

- Zoom
- Doxy
- Skype
- Google Meet
- Microsoft Team
- Other:

**Preface:**

The following questions assume that the patient can be seen via telehealth (e.g., the condition is appropriate, the patient has the necessary technology to receive treatment via telehealth, etc.).

1. Telehealth can be used (check all that apply)
  - As a substitute for in-person clinical visits
  - As an option to use in addition to in-person clinical visits.
2. In my professional opinion, telehealth is an effective platform for the delivery of OT services
  **Table:**
  SA	A	N	D	SD
3. Telehealth should be a treatment platform option for the delivery of OT services permanently for those conditions that can be treated successfully via telehealth?
  **Table:**
  SA	A	N	D	SD
4. I had fewer no-shows for telehealth visits than I usually have for in-person visits?
  **Table:**
  SA	A	N	D	SD
5. I was able to achieve established patient goals via telehealth
  **Table:**
  SA	A	N	D	SD
6. I achieved similar health outcomes using telehealth as I would have expected in person
  **Table:**
  SA	A	N	D	SD
7. I was able to be sufficiently productive using telehealth
  **Table:**
  SA	A	N	D	SD
8. Patients were satisfied with telehealth as a delivery platform
  **Table:**
  SA	A	N	D	SD	not applicable
9. Caregivers were satisfied with telehealth as a delivery platform
  **Table:**
  SA	A	N	D	SD	not applicable
10. I was satisfied with telehealth as a delivery platform
  **Table:**
  SA	A	N	D	SD
11. I would recommend telehealth as a service delivery platform to my friends and family members
  **Table:**
  SA	A	N	D	SD
12. I received reimbursement for telehealth services by (check all that apply):
  1. Medicare
  2. Medicaid
  3. Private Insurance
  4. Other _________________________________
13. I was able to effectively treat these diagnoses via telehealth (list up to three):
  ____________________________________________
  14) I was able to effectively use these interventions via telehealth (list up to three):
  ____________________________________________
  15) Is there anything else you would like to share regarding your experiences with telehealth and why you do or do not see telehealth as a viable treatment delivery platform?
  ____________________________________________
