# Errata

**DOI:** 10.5195/ijt.2021.6382

**Published:** 2021-06-22

**Authors:** Ellen R. Cohn

Correction to: Dahl-Popolizio, S., Carpenter, H., Coronado, M., Popolizio, N. J., & Swanson, C. (2020). Telehealth for the provision of occupational therapy: Reflections on experiences during the COVID-19 pandemic. *International Journal of Telerehabilitation, 12*(2), 77–92. https://doi.org/10.5195/ijt.2020.6328

In the article Dahl-Popolizio, S., Carpenter, H., Coronado, M., Popolizio, N. J., & Swanson, C. (2020). Telehealth for the provision of occupational therapy: Reflections on experiences during the COVID-19 pandemic. *International Journal of Telerehabilitation, 12*(2), 77–92. https://doi.org/10.5195/ijt.2020.6328, two references in the article metadata were listed incorrectly by the Editor:

“Author, (2014)” should be “Dahl-Popolizio, S., Loman, J., & Cordes, C. C. (2014). Comparing outcomes of Kinect video game-based occupational/physical therapy versus usual care. *Games for Health, 3*(3), 157–161. https://doi.org10.1089/g4h.2014.0002.”

“Author, (2017)” should be “Dahl-Popolizio, S., Rogers, O., Carroll, J., Muir, S., & Manson, L. (2017). Interprofessional primary care: The value of occupational therapy. *Open Journal of Occupational Therapy, 5*(3). https://doi.org/10.15453/2168-6408.1363.”

The metadata for the original article has been corrected.

